# Estimate of the magnitude of risky and protective behaviors associated with road traffic injuries in capitals participating in the Life in Traffic Project of Brazil

**DOI:** 10.1371/journal.pone.0275537

**Published:** 2022-10-19

**Authors:** Gabriela Silvério Bazílio, Rafael Alves Guimarães, José Ignacio Nazif-Munoz, Marie Claude Ouimet, Asma Mamri, Otaliba Libânio Morais Neto

**Affiliations:** 1 Instituto de Patologia Tropical e Saúde Pública, Universidade Federal de Goiás, Goiânia, Goiás, Brasil; 2 Faculdade de Enfermagem, Universidade Federal de Goiás, Goiânia, Goiás, Brasil; 3 Faculté de Médecine et des Sciences de la Santé, Université de Sherbrooke, Longueuil, Quebec, Canada; 4 Centre de recherche Charles-Le Moyne, Longueuil, Quebec, Canada; Southeast University, CHINA

## Abstract

**Background:**

Brazil occupies the fifth position in the ranking of the highest mortality rates due to RTI in the world. With the objective of promoting traffic safety and consequently reducing deaths, Brazil created the Life in Traffic Project (LTP). The main goal of LTP is reducing 50% of RTI deaths, by promoting interventions to tackle risk factors, such as driving under the influence of alcohol and excessive and/or inappropriate speed. Thus, the aim of this study was to estimate the magnitude of risky and protective factors for RTI in capitals participating in the LTP in Brazil. We estimated these factors according to sociodemographic (age group, sex, education, race and, type of road user).

**Methods:**

A total of 5,922 car drivers and motorcyclists from 14 Brazilian capitals participating in the LTP were interviewed. Data collection was carried out in sobriety checkpoints at night and consisted of the administration of an interview and a breathalyzer test. Risky and protective behaviors associated with RTI were investigated. Covariates of the study were: age, sex, education, race and, type of road user. Poisson multiple regression analysis was used to assess the relationship between variables of interest.

**Results:**

The prevalence of individuals with positive blood alcohol concentration (BAC) was 6.3% and who reported driving after drinking alcohol in the last 30 days was 9.1%. The others risky behaviors reported were: driving at excessive speed on roads of 50 km/h, using a cell phone for calls while driving, using a cell phone to send or read calls while driving, running a red light. Use of seatbelts and helmets showed prevalence above 96,0% Use of seatbelts showed prevalence of 98.6% among car drivers, and helmet use was described by 96.6% of motorcycle drivers. Most risky behaviors were more prevalent in younger age groups (except BAC measurement higher in older participants), in males (except for cell phone use), in participants with higher education level and without a driver’s license.

**Conclusion:**

Excessive speed and driving under the influence of alcohol, defined as priorities within the LTP, need more consistent interventions, as they still have considerable prevalence in the cities investigated. The factors described such as cell phone usage and passing red traffic lights should also need to be prioritized as a focus on promoting traffic safety.

## Introduction

In 2016, the World Health Organization (WHO) estimated that road traffic injuries (RTI) were responsible for 1.35 million deaths worldwide, representing the octave cause of death for all ages and the first in children and young adults aged 5 to 29 years old. RTI represent a serious public health problem, especially in low- and middle-income countries where mortality rates are three times higher than in high-income countries, concentrating 93% of deaths worldwide [1]. In addition to the significant impact on global mortality, RTI are responsible for multiple disabilities, decreased quality of life and high costs for health services [1–4].

Brazil also has a high burden of morbidity and mortality due to RTI. In 2016, the country occupied the fifth place in the ranking of countries with the highest mortality rates from RTI, behind India, China, the United States and Russia [1]. The WHO estimates, which include correction factors, have showed a mortality rate from RTI in Brazil of 19.7 deaths per 100,000 inhabitants in 2016 [1]. In 2019, data from the Ministry of Health’s Mortality Information System showed that there were 32,879 deaths due to RTI in Brazil [5]. The costs due to RTI in Brazil, especially related to the loss of productivity and health services are estimated at US$ 9 billion annually [6].

RTI are the results of a complex interaction of multiple factors and determinants related to individual behaviors, vehicles, society, and environment [7–9]. In terms of individual behaviors, the most related to increased risk in Brazil as well as worldwide, include driving under the influence of alcohol, driving at excessive and/or inappropriate speed, and distracted driving (eg cell phone use), and running red lights [10–15]. Other behaviors, such as the use of safety devices (eg use of seat belts for motor vehicles and helmets for motorcyclists) represent protective factors that can reduce the severity of RTI [8, 16]. The National Health Survey, conducted in 2019 in Brazil on individuals aged 18 years and over, reported data on RTI, driving under the influence of alcohol, and seat belt and helmet use [17, 18]. Results have shown that 2.4% of the respondents reported involvement in RTI in the previous 12 months and 17.0% reported driving a motor vehicle or riding a motorcycle after drinking alcohol in the last 12 months [17, 18]. Among car drivers, 20.3% reported irregular use of seat belts in the front seat when driving or while being a passenger; among motorcyclists, 17.4% reported irregular helmet use [18]. In terms of vehicles, society, and environment, Brazil has gone through a rapid and disorderly process of urbanization in recent decades. This process was accompanied by policies that subsidized the increase of the country’s motor vehicle and motorcycle fleet, to the detriment of the expansion and improvements of public transport, as well as the low investment in robust interventions for promoting the safety of urban roads and highways [1, 16, 19]. The high prevalence of risky behaviors, which contributes to the high magnitude of RTI in Brazil [10, 11], and the lack of interventions to promote traffic safety are core issues that need to be addressed.

A decade ago, Brazil started to implement new laws and interventions in relation to the WHO’s initiative through the United Nations that officially proclaim the period 2011–2020 as the “Decade of Action for Traffic Safety (2011–2020), with the global goal to reduce by half the total number of deaths caused by RTI, such as the zero-tolerance law for driving and drinking in drivers in 2008, 2012 and 2016 [20]. In 2010–2011, Brazil launched the Life in Traffic Project (LTP), an initiative coordinated by the Ministry of Health and the support of the Pan American Health Organization. The main goal of this project was to reduce traffic deaths by 50%. The project was initially implemented in five capitals: Palmas, Teresina, Belo Horizonte, Curitiba, and Campo Grande, each representing one of the five major regions of the country. From 2012 and 2013, the LTP was expanded to all Brazilian capitals and to municipalities with more than one million inhabitants. The actions of the LTP include traffic education; increased in the budget for the purchase of equipment for monitoring and inspection of driving under the influence of alcohol and excessive speed, in addition to an increase in inspection of these risk factors, among others. Although the initial program guidelines recommend focusing on the two risk factors (driving under the influence of alcohol and speeding), local programs in each municipality can develop actions focusing on other factors such as distracted driving, running red lights and the use of protective equipment. However, there is a gap in evidence of what actions are carried out in each municipality for these last factors [21–23].

Some studies have evaluated the degree of implementation and impact of LTP in reducing mortality and some risk factors for RTI [21, 23]. However, no nationwide study has assessed the magnitude of a set of risk and protective factors for RTI in representative samples of motor vehicle drivers and/or motorcyclists in capital cities that participated in the LTP in Brazil. In addition, estimates under distracted driving (for example, cell phone use while driving), driving at excessive speed and running red light are scarce in this country. Studies that use the breathalyzer tests to estimate the magnitude of driving under the influence of alcohol in drivers and the stratification of risk and protective factors for RTI according to sociodemographic variables (age group, sex, education, race, type of road user,) are limited. Thus, the aim of this study was to estimate the magnitude of risky and protective factors for RTI in capitals participating in the LTP in Brazil.

## Material and methods

### Setting

This cross-sectional study, assessing the magnitude of risk and protective factors for RTI, was carried out between March and December 2019 in 14 (51.8%) of the 27 Brazilian capitals participating in the LTP. In 2019, the estimated total population of the 14 capitals included in the study was 26,625,653 inhabitants [24].

The selection of participating capitals was carried out in two stages. The first included the five capitals that implemented the LTP in 2010 and 2011, located in their respective major regions (Belo Horizonte [Southeast region]; Campo Grande [Midwest region], Curitiba [South region], Palmas [North region], and Teresina [Northeast region]). In the second stage, simple random sampling was performed to select the nine capitals that implemented the LTP in its expansion phase in 2012 and 2013 [21]. The second stage of the selection took place in pairs to reach the same large geographic regions of the five capitals that started the LTP: Boa Vista and Macapá (North region), Salvador and São Luís (Northeast region), Goiânia and Cuiabá (Center-West region), Florianópolis (South Region), Vitória and São Paulo (Southeast Region). Thus, the survey included a representative sample of the target population covering all capitals in the large regions of Brazil.

### Participants

Individuals aged 18 years or over and residents of private permanent households in one of the capitals selected for the survey were considered eligible. Exclusion criteria were driving a commercial vehicle (eg taxi, motorcycle taxi, bus, UBER, delivery vehicle) or a heavy vehicle (eg truck, van, bus). Participants also did not need to have legal registration (driver’s license) to drive vehicles for inclusion in the study. They did not receive compensation for their participants.

### Sampling

A two-stage cluster sampling plan was carried out in this study. The first stage of sampling consisted of choosing sobriety checkpoints in each selected capital, thereby drivers would be stopped and invited to participate in the study. The choice of approach locations, was made by the research team, included public roads with more intense traffic and/or with a greater concentration of bars, restaurants, and nightclubs.

In the second stage of sampling, car drivers and motorcyclists were selected. At this stage, a systematic random sampling of cars and motorcycles was carried out at the sobriety checkpoints, with one car or motorcycle being selected every three or five, depending on the flow of the local road.

The sample was stratified, for each city, according to the type of road user (car drivers or motorcyclists), considering the proportion of the vehicle fleet for each city. The sample calculation was performed considering the main risk factor of interest in the study as a reference (positive blood alcohol test). The sample size was calculated assuming a 95% confidence interval (95.0% CI; α = 0.05) and 2.0 design effect for complex samples [25]. A mean prevalence of positivity in the breathalyzer test or report of driving under the influence of alcohol in the last six hours of 10.2% was used as a reference for sample calculation based on a previous study conducted in 3,398 drivers randomly selected from all regions of Brazil [26]. To the base sample calculation (n = 282) an expected rejection rate of 20.0% was added, resulting in a minimum required sample of 338 individuals for each selected capital, resulting in an estimated minimum total sample of 4,732 participants.

### Measures

Data collected included the blood alcohol concentration (BAC), risk and protective factors for RTI, and demographic characteristics. BAC was measured in mg/L using a breathalyzer test.

The frequency of risky and protective driving behaviors associated with RTI were asked to the participants (S1 Appendix). Responses were on a 5-point Likert scale ranging from “1” never to “5” always. The following behaviors while driving was probed: driving at excessive speed on roads of 50km/h, cell phone use (phone calls), cell phone use (texting), running red lights, regular use of seat belts, and regular helmet use. Driving after drinking alcohol in the last 30 days was evaluated with dichotomous responses such as “0” no or “1” yes for this behavior.

Different denominators were used: i) the total number of drivers who performed the breathalyzer test for the BAC; ii) the total number of car drivers interviewed for driving after drinking alcohol, speed, cell phone use and running red lights; iii) the total number of car drivers or motorcyclists interviewed for seat belt use; and iv) the total number of motorcyclists interviewed for helmet use.

The following demographic variables were used to stratify the indicators: age group (18 to 29 years old, 30 to 39 years old, 40 to 49 years old, 50 to 59 years old or 60 years old or more) [27]; sex (male or female); education (never studied/incomplete elementary school, complete elementary/incomplete middle school, complete middle school/incomplete higher education or complete higher education or more); self-reported race (White, Black, Mixed race or Others, eg, Asian or Indigenous); have a driver’s license (yes or no) and type of road user (car driver or motorcyclist).

### Procedures

Data were collected from Wednesday to Sunday, from 9:00 pm to 2:00 am. Interviewers were rigorously trained to application of the collection instrument and performance of the breathalyzer test. Police officers or traffic agents were co-trained, as these individuals were responsible for the initial approach to drivers at collection points, ensuring the safety of the research team in data collection [26]. A pilot test on 1% of the estimated sample was carried out to analyze the content of the collection instrument and standardize the study methods.

**Fig 1:**
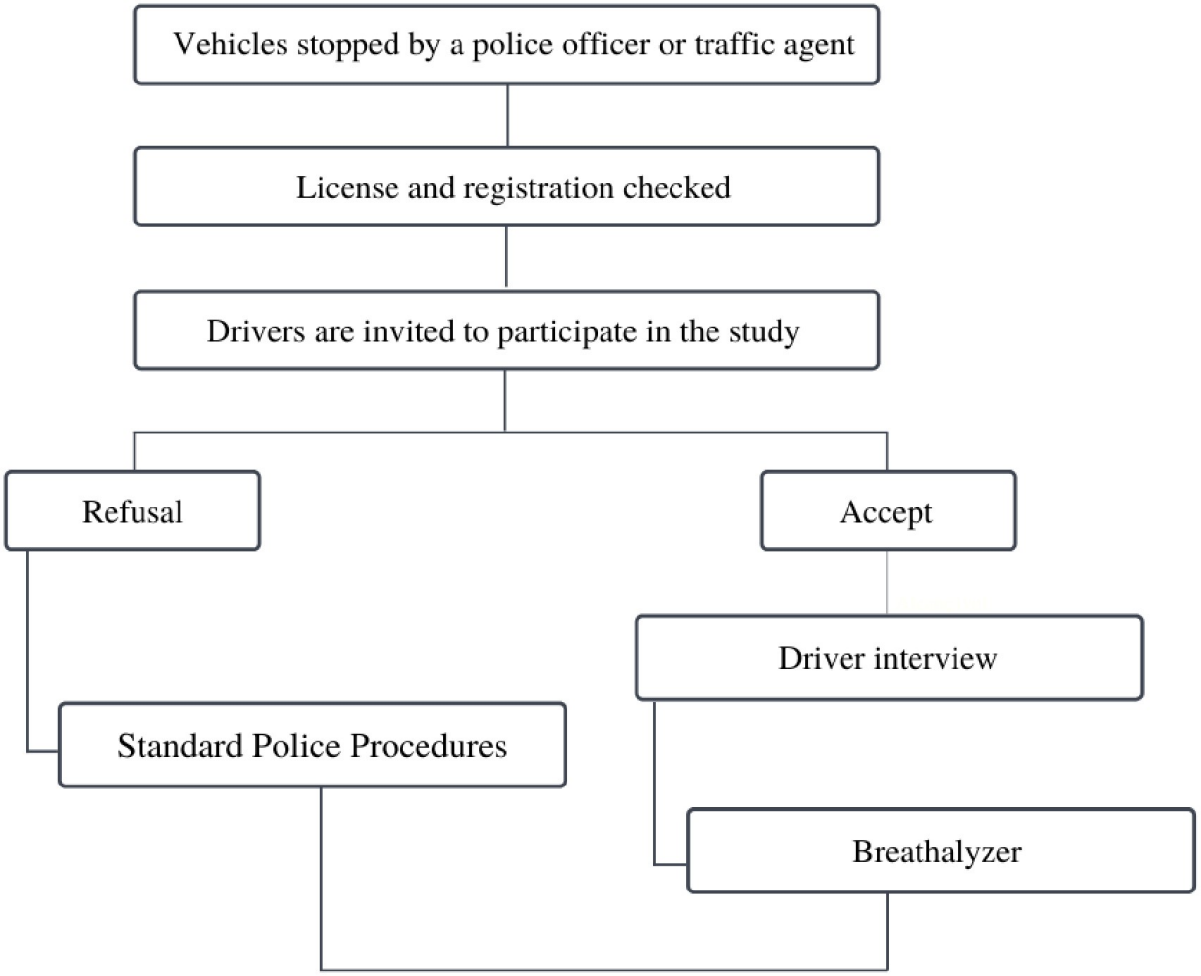
Study data collection flowchart, 2019.

The study data collection procedure is shown in Fig 1. Specifically, each selected vehicle was stopped by a police officer or traffic agent who acted in road traffic inspection at the municipal, state, or federal levels of each city. After checking the driver’s license and registration, the police or traffic inspection agents informed drivers about the study and invited them to participate. Conductors who agreed to participate were referred to the interview team in a parking lot away from the team of police and traffic agents who made the initial approach. This procedure minimized the possibility of response bias.

After clarification of the objectives and methods of participation in the study by the research team, all drivers who agreed to participate signed the Informed Consent Form and were interviewed using a structured instrument on demographic and risky and protective behaviors associated with RTI. The interviews were conducted on tablets, properly programmed with the study questionnaire, and operating online or offline. Next, the participant was invited to perform the active breathalyzer test, which used equipment validated and approved by INMETRO (BAF-300 ELEC). In case of acceptance of the test, a trained operator explained to the driver the functioning and methods of the instrument. The results of the breathalyzer test were recorded exclusively on the study form and the data were not shared with the police or traffic agent, nor with the participating driver. After carrying out the study’s breathalyzer test, participants were referred to the police or traffic agent to perform standard procedures.

### Statistical approach

A descriptive analysis of the indicators was carried out using relative frequencies and respective 95%CI for the total sample and stratified by survey capital. Next, for the total sample, the indicators were estimated according to a group of demographic characteristics (age group, sex, education, race, and type of road user). The Poisson multiple regression adjusted for demographic characteristics was applied with the calculation of Adjusted Prevalence Ratios (aPR) and respective 95%CI to establish potential associations [28]. The independent variables showed no evidence of multicollinearity (tetrachoric correlation coefficients between 0.10 and 0.32) for Poisson models; in addition, the goodness-of-fit chi-squared test showed that the Poisson models showed a good fit (p-value>0.05). In addition, our sample was relatively large for carrying out the Poisson model. Values of p<0.05 were considered statistically significant. All analyses used sampling weights to adjust for variation in the probability of selection and were performed using STATA 16.0 (“survey”) [29]. More specifically, weights were used to ensure the representativeness of the survey, bringing the sample demographic distribution closer to that estimated for the reference population of drivers. Weights were obtained through mathematical procedures using population distribution variables, such as age and sex collected in this survey and the reference population. To calculate the weights, the reference population of drivers in Brazil collected in the 2013 National Health Survey was used [11]. The method used to build the weights was the rake using the age and sex variables of the drivers [30].

### Ethical aspects

This study was approved by the Ethics Committee for Research with Human Beings of the Federal University of Goiás, registration number 2,854,899/2018. Written consent was obtained from all participants.

## Results

A total of 6,088 drivers were selected to compose the survey sample, of which 5,922 agreed to participate, which represented an acceptance rate of 97.3%. In addition, the acceptance rate of performance in the breathalyzer test was high (96.8%; n = 5,735). S1 Table shows the percentage of drivers who accepted to participate in the interview and the percentage of participants who agreed to take the by capital.

The mean age of participants was 38.0 years, with 29.4% aged 18–29 years, 28.2% between 30–39 years, with 18.3% aged 40–49 years, with 6.8% aged 50–59 years and with 4.7% aged 60 years or over. Of the total, the majority (65.4%) were male, car drivers (76.3%) and have a driver’s license (98.0%). As for education, 4.0% of the sample had never studied/had incomplete elementary education; 7.1% complete elementary/incomplete middle school; 46.0% had completed middle school/incomplete higher education and 42.9% had completed higher education or more. About self-reported race, 42.8% were white, 42.3% mixed race, 12.4% black and 2.6% others. In the sample, 2.0% of the drivers interviewed did not have a legal record to drive.

Table 1 shows the prevalence of the eight risky and protective behaviors associated with RTI, stratified by capital. The prevalence of individuals with positive BAC was 6.3%, ranging from 3.1% in Vitória to 10.9% in Boa Vista. Those who reported driving after drinking alcohol in the last 30 days was 9.1%. The risky behaviors most reported were driving at excessive speed on roads of 50 km/h (46.7%), using a cell phone for calls while driving (26.1%), using a cell phone to send or read calls while driving (24.2%), running a red light (14.7). Finally, most motorcyclists reported using a helmet (98.6%) and most car drivers reported using their seat belt (96.6%).

**Table 1:** Prevalence of risky and protective behaviors associated with traffic crashes in the 14 Brazilian capitals participating in the Life in Traffic Project, 2019.

**City**	**n**	**Drivers with positive BAC**	**Drivers who reported driving after drinking alcohol in the last 30 days**	**Drivers who reported the habit of driving at excessive speed on roads of 50km/h**	**Drivers who reported using a cell phone to make calls while driving**
		**% (95% CI)**	**% (95% CI)**	**% (95% CI)**	**% (95% CI)**
Belo Horizonte	380	4.2 (2.4; 7.4)	6.2 (4.0; 9.6)	49.4 (43.9; 55.0)	31.4 (26.5; 36.8)
Boa Vista	457	10.9 (7.8; 15.0)	12.3 (8.7; 17.1)	48.0 (42.3; 53.8)	27.3 (22.9; 32.1)
Campo Grande	355	7.6 (4.9; 11.6)	12.3 (9.1; 16.6)	64.9 (58.6; 70.7)	28.8 (23.4; 34.7)
Cuiabá	408	8.4 (5.6; 12.4)	9.2 (6.8; 12.4)	52.6 (46.5; 58.5)	21.6 (17.3; 26.7)
Curitiba	341	5.8 (3.6; 9.1)	10.2 (7.4; 14.1)	52.6 (46.6; 57.5)	32.0 (26.9; 37.5)
Florianópolis	364	4.9 (3.0; 8.1)	9.1 (6.6; 12.4)	49.9 (42.8; 55.1)	19.5 (15.3; 23.8)
Goiânia	329	3.4 (1.7; 6.7)	6.1 (3.8; 9.8)	53.5 (47.3; 59.6)	31.8 (26.6; 37.5)
Macapá	581	3.6 (2.2; 5.8)	5.8 (3.9; 8.6)	39.5 (34.5; 44.7)	21.6 (18.1; 25.6)
Palmas	469	10.0 (7.4; 13.4)	14.0 (11.0; 17.1)	45.9 (41.1; 50.9)	21.8 (18.1; 26.0)
Salvador	383	5.1 (3.1; 8.4)	3.9 (2.4; 6.2)	28.3 (23.3; 33.8)	26.1 (21.7; 31.0)
São Luís	513	5.6 (3.8; 8.2)	13.3 (10.3; 17.1)	48.9 (43.9; 54.1)	30.9 (26.8; 35.4)
São Paulo	440	7.9 (5.4; 11.2)	8.2 (5.7; 11.6)	30.8 (25.6; 36.5)	24.6 (20.1; 29.7)
Teresina	488	8.5 (6.2; 11.7)	12.6 (9.9; 16.0)	34.4 (29.8; 39.5)	22.4 (18.6; 26.8)
Vitória	414	3.1 (1.7; 5.8)	2.7 (2.1; 6.6)	56.3(50.8; 61.7)	25.8 (21.5; 30.6)
**Total**	**5.922**	**6.3 (5.7; 7.1)**	**9.1 (8.3;9.9)**	**46.7 (45.2; 48.3)**	**26.1 (24.8; 27.4)**
**City**	**n**	**Drivers who reported using a cell phone to send or read messages while driving**	**Drivers reported the habit of running red light**	**Motorcyclists who reported regular helmet use**	**Car drivers who reported regular use of seat belts**
		**% (95% CI)**	**% (95% CI)**	**% (95% CI)**	**% (95% CI)**
Belo Horizonte	380	28.5 (24.0; 33.6)	14.7 (11.0; 19.2)	96.1 (85.3; 99.0)	97.0 (94.2; 98.5)
Boa Vista	457	22.4 (18.3; 30.0)	13.3 (9.7; 18.0)	100.0	94.6 (91.3; 96.7)
Campo Grande	355	33.5 (27.8; 39.7)	22.5 (17.9; 28.0)	100.0	95.4 (91.5; 97.6)
Cuiabá	408	20.7 (16.4; 25.6)	23.4 (18.9; 28.8)	100.0	99.0 (97.4; 99.3)
Curitiba	341	32.9 (27.8; 38.3)	12.6 (9.4; 16.8)	100.0	98.4 (96.4; 99.3)
Florianópolis	364	22.3 (17.9; 27.4)	10.6 (7.3; 15.1)	100.0	96.3 (93.5; 97.9)
Goiânia	329	35.5 (30.0; 41.3)	20.3 (15.9; 25.5)	95.3 (82.9; 98.9)	97.3 (94.4; 98.7)
Macapá	581	15.1 (12.2; 18.7)	10.9 (8.3; 14.1)	100.0	96.1 (94.0; 97.6)
Palmas	469	17.5 (14.2; 21.3)	7.2 (5.0; 10.1)	100.0	94.9 (91.7; 96.8)
Salvador	383	23.6 (19.6; 28.3)	12.4 (9.1; 16.6)	94.2 (68.7; 99.2)	98.8 (97.3; 99.5)
São Luís	513	22.8 (19.2; 26.8)	14.5 (11.3; 18.5)	98.7 (91.3; 99.8)	94.6 (92.1; 96.3)
São Paulo	440	26.4 (21.8; 31.6)	6.6 (4.4; 9.9)	100.0	96.4 (93.6; 98.0)
Teresina	488	17.4 (14.1; 21.4)	23.4 (19.4; 27.8)	95.2 (90.8; 97.6)	94.4 (91.0; 96.6)
Vitória	414	19.9 (16.0; 24.4)	13.0 (9.9; 16.9)	100.0	97.3(95.2; 98.5)
**Total**	**5.922**	**24.2 (23.0; 25.4)**	**14.7 (13.6; 15.8)**	**98.6 (97.4; 99.2)**	**96.6 (96.0; 97.1)**

BAC: Blood Alcohol Concentration; 95% CI: 95% Confidence Interval.

Table 2 shows the prevalence of the risky behaviors evaluated in the set of 14 capitals adjusted by age group, sex, education, race, driver’s license and type of road user.

**Table 2:** Prevalence of risky behaviors associated with traffic crashes in the 14 Brazilian capitals participating in the life in traffic project and adjusted prevalence ratio by age, sex, education, race and type of road user, 2019.

**Variables**	**% Drivers with positive BAC**	**aPR**	**p-value** *	**% Drivers who reported driving after drinking alcohol in the last 30 days**	**aPR**	**p-value** *	**% Drivers who reported the habit of driving at excessive speed on roads of 50km/h**	**aPR**	**p-value** *
**Age (years)**									
≥ 60	9.7 (6.4–14.4)	1.00		7.4 (4.8–11.0)	1.00		31.4 (25.6–37.8)	1.00	
50–59	8.6 (6.3–11.8)	0.91 (0.55–1.52)	0.719	7.1 (5.2–9.6)	1.09 (0.65–1.82)	0.751	37.8 (33.5–42.4)	1.22 (0;97–1.52)	0.090
40–49	6.1 (4.7–7.9)	**0.60 (0.37–0.98)**	**0.039**	8.3 (0.7–10.3)	1.24 (0.78–1.97)	0.365	42.3 (38.9–45.7)	**1.38 (1.12–1.71)**	**0.003**
30–39	6.2 (5.2–7.4)	**0.62 (0.39–0.99)**	**0.045**	10.3 (8.9–11.9)	1.53 (0.99–2.38)	0.054	47.8 (45.3; 50.4	**1.56 (1.28–1.92)**	**<0.001**
18–29	5.1 (4.1–6.2)	**0.47 (0.29–0.75)**	**0.002**	9.7 (8.3–1.3)	1.49 (0.96–2.31)	0.076	56.2 (54.6; 58.7)	**1.88 (1.53–2.30)**	**<0.001**
**Sex**									
Female	4.3 (3.2; 5.8)	1.00		6.2 (4.9; 7.9)	1.00		43.4 (40.2; 46.5)	1.00	
Male	7.4 (6.6; 8.3)	**1.68 (1.20; 2.36)**	**0.002**	10.6 (9.6 11.6)	**1.89 (1.47; 2.44)**	**<0.001**	48.5 (46.9; 50.2)	**1.16 (1.07; 1.26)**	**<0.001**
**Level of education**									
Never studied/incomplete elementary school	10.3 (6.3; 16.4)	1.00		7.2 (4.2; 11.7)	1.00		37.3 (30.6; 44.6)	1.00	
Complete Elementary/Incomplete Middle School	7.3 (4.8; 10.8)	0.91 (0.51; 1.62)	0.409	7.5 (5.1; 10.9)	1.06 (0.57; 1.97)	0.849	47.2 (41.6; 52.9)	1.14 (0.91–1.473	0.241
Complete middle school/incomplete higher education	6.4 (5.4; 7.6)	1.03 (0.65; 1.65)	0.901	8.7 (5.1; 9.9)	1.32 (0.79; 2.24)	0.291	46.9 (44.7; 49.2)	1.08 (0.89–1.31)	0.451
Complete higher education or more	5.8 (4.8; 6.9)	0.97 (0.60; 1.55)	0.882	9.9 (8.7; 11.3)	**1.77 (1.04; 3.00))**	**0.035**	47.3 (45.0; 49.6)	1.18 (0.97–1.43)	0.092
**Race**									
Mixed race	6.4 (5.4; 7.5)	1.00		9.2 (8.0; 10.5)	1.0		45.4 (43.1; 47.7)	1.00	
White	5.9 (4.9; 7.1)	0.95 (0.74–1.23)	0.706	8.9 (7.7; 10.2)	0.99 (0.81; 1.20)	0.820	49.0 (46.7; 51.4)	**1.12 (1.05–1.21)**	**0.001**
Black	7.6(5.5; 10.4)	1.09(0.78–1.55)	0.595	9.4 (7.2; 12.1)	0.99 (0.75; 1.33)	0.982	44.5 (40.2; 48;9)	0.99 (0.89–1.10)	0.853
Others	8.5 (4.6; 15.2)	1.33 (0.72–2.48)	0.353	7.4 (3.6; 13.3)	0.80 (0.43; 1.47)	0.475	42.0 (32.7; 48.9)	0.93 (0.74–1.18)	0.569
**Driver’s license**									
Yes	6.0 (5.4–6.8)	1.00		8.9 (8.2–9.8)	1.00		46.9 (45.4–48.4)	1.00	
No	21.6 (13.6–32.5)	**4.1 (2.53–6.63)**	**>0.001**	14.2 (8.3–23.2)	**1.91 (1.08–3.38)**	**0.026**	38.7 (28.6–49.9)	0.79 (0.59–1.05)	0.105
**Type of road user**									
Motorcyclist	7.0 (5.5; 9.0)	1.00		9.2 (7.5; 11.4)	1.00		49.0 (45.5;52.6)	1.00	
Car driver	6.1 (5.4–7.0)	1.01 (0.73–1.38)	0.966	9.0 (8.2–9.9)	1.03 (0.80–1.33)	0.791	46.0 (44.4; 47.7)	0.96 (0.88–1.05)	0.393
**Variable**	% **Drivers who reported using a cell phone to make calls while driving**	**aPR**	**p-value** *	% **Drivers who reported using a cell phone to send or read messages while driving**	**aPR**	**p-value** *	% **Drivers reported running red lights**	**aPR**	**p-value** *
**Age (years)**									
≥ 60	13.2 (9.6–18.0)	1.00		6.6 (4.2–10.5)	1.00		10.6 (7.3–15.2)	1.00	
50–59	21.6 (18.1–25.6)	**1.7 3(1.21–2.47)**	**0.003**	14.4 (11.5–17.8)	**2.40 (1.42–4.04)**	**0.001**	13.3 (10.4–16.8)	1.26 (0.86–1.95)	0.311
40–49	22.9 (20.3–25.8)	**2.00 (1.42–2.80)**	**<0.001**	19.7 (17.2–22.5)	**3.61 (2.20–5.91)**	**<0.001**	15.7 (13.4–18.3)	1.46 (0.98–2.18)	0.064
30–39	27.8 (25.6; 30.0)	**2.47 (1.80–3.42))**	**<0.001**	27.5 (25.3–29.8)	**5.17 (3.19–8.39)**	**<0.001**	13.9 (12.2–15.7)	1.30 (0.88–1.92)	0.188
18–29	31.5 (29.2; 33.8)	**3.12 (2.26–4.31)**	**<0.001**	32.2 (29.9–34.6)	**6.87 (4.24–11.14)**	**<0.001**	16.0 (14.1; 18.0)	**1.50 (1.02–2.22)**	**0.040**
**Sex**									
Female	27.8 (25.1; 30.4)	1.00		27.8 (25.3; 30.5)	1.00		12.5 (10.6; 14.8)	1.00	
Male	25.2 (23.9; 26.6)	1.10 (0.99;1.23)	0.067	22.2 (21.0; 23.5)	1.01 (0.90–1.12)	0.868	15.8 (14.6; 17.0)	**1.22 (1.01; 1.48)**	**0.040**
**Level of education**									
Never studied/incomplete elementary school	10.9 (7.4; 15.7)	1.00		9.7 (6.4; 14.5)	1.00		14.6 (10.2; 20.6)	1.00	
Complete Elementary/Incomplete Middle School	18.4 (14.6; 22.9)	1.49 (0,98; 2.27)	0.063	15.3 (11.9; 19.5)	1.28 (0.82; 2.03)	0.281	18.8 (14.9; 23.4)	1.25 (0.24; 1.91)	0.303
Complete middle school/incomplete higher education	24.3 (22.5; 26.2)	**1.63 (1.13; 2.36)**	**0.010**	21.7 (20.0; 23.5)	1.37 (0.91; 2.04)	0.130	14.9 (13.4; 16.6)	0.99 (0.67; 1.46)	0.979
Complete higher education or more	30.4 (28.4 32.5)	**1.80 (1.24; 2.60)**	**0.002**	29.3 (27.3; 31.3)	**1.64 (1.10; 2.45)**	**0.016**	13.8 (12.3; 15.4)	1.01 (0.69; 1.50)	0.941
**Race**									
Mixed race	24.2 (22.4; 26.1)	1.00		22.0 (20.3; 23.9)	1.00		15.1 (13.6; 16.8)	1.00	
White	29.2 (27.1; 31.3)	1.06 (0.96–1.18)	0.212	28.1 (26.2; 30.3)	**1.14 (1.03–1.26)**	**0.011**	13.8 (12.3; 15.5)	0.96 (0.82–1.13)	0.652
Black	21.6 (18.6; 25.0)	0.94 (0.80–1.10)	0.415	17.7 (15.0; 20.8)	0.85 (0.72–1.01)	0.070	16.3 (13.5; 16.8)	1.07 (0.86–1.33)	0.560
Others	25.4 (17.8; 34.8)	1.04 (0.73–1.47)	0.841	23.4 (15.8; 33.3)	1.05 (0.73–1.51)	0.801	14.6 (8.6; 24.0)	0.99 (0.58–1.68)	0.967
**Driver’s license**									
Yes	26.4 (25.1–27.7)	1.00		24.4 (23.2–25.7)	1.00		14.7 (13.7–15.8)	1.00	
No	12.5 (7.4–20.4)	0.86 (0.53–1.39)	0.542	12.7 (5.5–20.1)	0.93 (0.58–1.49)	0.754	12.2 (6.9–20.8)	0.73 (0.40–1.33)	0.306
**Type of road user**									
Motorcyclist	8.4 (6.8; 10.4)	1.00		7.4 (5.9; 9.3)	1.00		16.4 (13.9; 19.1)	1.00	
Car driver	31.6 (30.1; 33.1)	**3.92 (3.14; 4.91**	**<0.001**	29.4 (27.9; 30.9)	**4.13 (3.25; 5.25**)	**<0.001**	14.1 (13.0; 15.3)	0.93 (0.77; 1.13)	0.462

aPR = Adjusted prevalence ratio.

*Wald test.

Note: models adjusted for age, sex, level of education, race and type of road user.

The prevalence of self-report driving after consumption of alcoholic beverages in the last 30 days was higher in males and without driver’s license. While the prevalence of positive BAC was also higher in males, conductors without driver’s license and it was higher in individuals aged 50 years or more (Table 2).

Excessive speed on speed lanes of 50 km/h was higher in participants in the 18–49 year-old group, in males and who were Whites. No differences were found for type of road user (Table 2).

The prevalence of cell phone use for calls or read/send messages while driving was higher in participants in the 18–59-year-old age group compared to older road users, in those with higher education compared to those with never studied or incomplete elementary school, and in car drivers compared to motorcyclists. The prevalence of cell phone use for read/send messages was also higher in White participants compared to mixed race participants. No differences were found between males and females (Table 2).

The prevalence of drivers who reported the running red lights was higher in males than in females and in participants in the 18–29-year-old age. No differences were found for education, race, and type of road user (Table 2).

## Discussion

This study provided estimates of the prevalence of important risky and protective behaviors associated with RTI in 14 Brazilian capitals participating in the LTP. In addition, prevalence of risky behaviors were compared by age, sex, education, race, driver’s license and type of road user. Using a method of collection at sobriety checkpoints, results showed that 6.3% of drivers were positive for the breathalyzer test. In addition, 9.1% reported driving under the influence of alcohol in the last 30 days. About half of the sample reported excessive speed on roads with a maximum speed of 50 km/h, approximately a quarter reported using their cell phone for calls and/or messages while driving, and about one sixth reported running red lights. The use of protective equipment (safety belt and helmet) was highly prevalent. Most risky behaviors were more prevalent in younger age groups (except BAC measurement higher in older participants), in males (except for cell phone use), and in participants with higher education level. White participants had a higher prevalence of cell phone use for messages while driving and reported excessive speed when compared to multiracial individuals. Drivers without a driver’s license had a higher prevalence of BAC and driving after alcohol use.

One of the main risk factors directly associated with the occurrence of crashes is driving after drinking alcohol. The prevalence of drivers with positive BAC in the current study was 6.3%. Surveys conducted at sobriety checkpoints in other countries showed positive prevalence BAC ranging from 4.2% to 12.0% in Canada and the United States [31–33]. In this survey, 9.1% of drivers reported driving after drinking alcohol in the last 30 days. This percentage is lower than those reported in two surveys conducted in Brazil that used the same question, but with different sampling methodologies: the 2019 National Health Survey [18] and the 2018 Surveillance of Risk Factors and Protection for Chronic Diseases by Telephone Survey [34]. The surveys collected data through household and telephone interviews, respectively, and found higher prevalence (i.e., 17,0% and 11,4%) than the current study. The results of the current study appear more similar to those found in surveys conducted in other countries, such as the United States with 8.5% [35] and Spain with 9.7% [36].

Driving under the influence of alcohol (self-report and positive BAC) was statistically associated with male sex. Men have a higher prevalence of alcohol use and alcohol abuse, as well as a lower perception of risk and some cultural factors (for example, more aggressiveness), which increases the likelihood of engaging in risky behavior such as driving under the influence of alcohol when compared to women [37–39]. Positive BAC associated with the increase in age was also another finding, as well as another study conducted in Brazil in sobriety checkpoints [37]. Studies conducted in more developed countries have shown a greater positivity of BAC in young people when compared to older adults [32, 40, 41]. In Brazil, it is possible that the characteristics of drivers who travel at night have influenced the result. There was a greater proportion of older individuals in the sample when compared to younger ones. In the country, older individuals have greater access to vehicles than younger people, which increase the probability of engaging in this behavior [42]. It is even possible that the younger individuals in this study use more transport apps (eg: UBER) to get around after drinking alcohol than the older ones. However, further studies are needed to explain the effect of age on the prevalence of BAC in Brazil.

The significant association of the higher prevalence of driving after alcohol use and drivers without legal registration is an unprecedented finding in Brazil. This may demonstrate the fragility of actions to inhibit this practice and to provide the population with expanded access to the necessary preparation to obtain the registration.

Brazil is among the countries with strict legislation on drinking and driving, the Dry Law launched in 2008 and reformulated in 2012 and 2016, established zero tolerance limits for alcohol for drivers and increased inspections and penalties [43, 44], but many drivers persist with this habit. Probably due to a lack of law enforcement in several Brazilian cities. In addition, the laws are recent, and this practice may be related to the cultural characteristics of the population and enforcement after de dry law as well the educational efforts still need to be made so that the prevalence decreases.

For the indicator driving at excessive speed on roads of 50km/h, 46.7% of drivers reported this behavior, with higher prevalence in males and individuals in the younger age groups and whites. Excessive speed, in addition to being an important crash predictor, is also directly related to RTI [45]. Studies show that driving above speed limits, increases the risk of a collision [45, 46]. This high prevalence and association with male sex and younger population was also evidenced in other countries more developed [47, 48].

Cell phone use for both making calls and reading/sending messages while driving was described by approximately 25% of drivers. No studies were found using the same methodology that investigated this indicator in Brazil and international studies investigating the prevalence reported by drivers are also scarce. However, other surveys conducted in the United States [49] and Ukraine [50] have also described similar frequencies of this behavior (18.7% and 28.2%, respectively). In Brazil’s legislation, the use of cell phones while driving is strictly prohibited, but this study has shown that it is adopted by many drivers, especially younger ones, with a higher level of education and white participants. Cell phones used for calls and internet access in 94.0% of Brazilian households, mainly by younger people, which may explain the higher prevalence of cell phone usage while driving by young people [51]. In addition, young people are less likely to understand the risks associated with this behavior [52]. Results have also shown that individuals who declared themselves White had a higher prevalence of using cell phones to read and send messages while driving; in addition to the association between higher education level and the two indicators of cell phone use and driving evaluated. Previous studies found that White students were more likely to report texting while driving [53, 54]. Other investigations also showed a higher prevalence of cell phone use while driving in individuals with higher level of education [53]. Few studies analyzed differences in the prevalence of cell phone use for sending/reading messages and driving among racial groups. In Brazil, it is known that race and education are directly associated with income. There is a positive correlation between whites or those with higher education and higher incomes [55]. Consequently, this contributes to access inequalities for both cell phone use and vehicle use which may explain the greater probability of this behavior for this population.

Running a red traffic light is an important risk factor for serious collisions, and road crossings are one of the main places of occurrence of crashes caused mainly by not respecting traffic signs [15]. In this study, the prevalence of the drivers reported the habit of running red traffic lights was 14.7%, with higher in men than women and youngers (18–29 years). This behavior is associated with individual characteristics such as impatience, haste and a sense of impunity [56]. Thus, traffic inspection is an important ally to reduce this practice. The use of traffic light cameras can drastically reduce this habit, increasing surveillance [57]. In Brazil, this feature needs to be installed consistently to increase surveillance of this behavior, facilitating the application of penalties and, consequently, reducing the prevalence of this habit.

The results of the study show that drivers from the capitals participating in the LTP adhere to traffic legislation about protective equipment, such as seat belts and wearing a helmet. The estimates of seat belt use among car drivers (96.6%) in the current study was higher than the estimates of the 2019 National Health Survey in Brazil with a prevalence of regular seat belt use in urban areas of 82.6% [18]. It was also higher than use reported or observed in other countries, such as the United States (87.0%) [58] and Spain (70.8%) [59].

For motorcyclists, reported use of helmets in the current study (98.0%) was higher than the estimates of the 2019 National Health Survey in Brazil showing 89.2% of regular use in urban area residents [18]. Reported use in Brazil from these two studies suggest, however, higher use than in other countries with similar motorcycle use, such as Thailand (43.7%) [60] and Índia (44.5%) [61].

The differences found regarding the estimates of the use of protective equipment between this study and the 2019 PNS maybe due to methodological and sample representative differences. The present survey was conducted in 14 Brazilian capitals at sobriety checkpoints. Although the data collection was conducted away from the police team, participants may have underreported their behaviors, while the 2019 National Health Survey was conducted in all Brazilian states, capitals and non-capitals and the data collection was carried out in households. The results of this study suggest that seat belt and helmet use have reached a very good level of implementation in the Brazilian population and some factors, such as the law, such as the Brazilian traffic code and may have played a role. Other studies, such as road observations, would provide more information on the prevalence of these protective device in the population.

The present study has some limitations. First, the cross-sectional nature does not allow the establishment of temporality and, consequently, causality between the independent demographic variables and the analyzed indicators. Also, it also does not allow establishing causality between the applications of traffic safety legislation in Brazil and the LTP and the results found. Second, the study included a lower proportion of motorcyclist and women, which may raise concerns about the representativeness of these subgroups in the survey. To alleviate possible sample distortions, the use of the complex sample module with calculations of post-stratification weights based on a representative sample of Brazilian drivers made it possible to minimize this bias in the study. Third, except for the objective measurement of the BAC with the breathalyzer test, most factors were based on self-report that are subjective measures and subject to participant response bias, especially to sensitive questions such as a history of drinking and driving. However, participants were assured that their data collected in a place away from the police or traffic agents would not be shared with them.

Despite the limitations, this study is a pioneering study with a representative sample of drivers from 14 capitals distributed in the five Brazilian regions, which included, in addition to an interview, the objective measurement of blood alcohol levels. The data can be analyzed and used as a source of information on the behavior of Brazilian drivers and motorcyclists and support the monitoring, evaluation and planning of public traffic safety policies.

In conclusion, the risky factors defined as priorities for interventions under the LTP, driving under the influence of alcohol and driving at excessive speed had considerable prevalence, need more forceful interventions aimed at reducing them. In addition, the other factors described such as cell phone use and passing red traffic lights also need to be prioritized, as they also had high magnitude. Driving distracted using cell phones represents one of the biggest challenges faced by the difficulty of inspection and the total insertion of this equipment in daily life with features that go beyond the use for messages and calls. The survey showed that some groups (eg. Male sex, adult more old, white individuals and with higher education) should be prioritized for the direction of traffic education and inspection policies by risk factor, with an emphasis also on capitals with higher prevalence of the main factors associated with traffic crashes. And finally, the need to intensify inspections and expand access to eliminate the presence of drivers without proper legal registration in traffic.

## Supporting information

S1 Table Number of sampled conductors and acceptance rate (TA) of participation in the study. (DOCX)

S1 Appendix Questionnaire. (DOCX)

S2 Appendix Dataset. (DTA)
