# Supplementary material for: Estimate of the magnitude of risky and protective behaviors associated with road traffic injuries in capitals participating in the Life in Traffic Project of Brazil
Source: PLoS One. 2022 Oct 19;17(10):e0275537. doi: 10.1371/journal.pone.0275537 (PMC9581410; doi:10.1371/journal.pone.0275537)
Supplement: S1 Table — (DOCX) [file pone.0275537.s001.docx]

**Supporting Information**

**Table S1.** Number of sampled conductors and acceptance rate (TA) of participation in the study

| **City** | **Interview** | | | **Breathalyzer test** | |
| --- | --- | --- | --- | --- | --- |
|  | **N** | **n** | **AR1 (%)** | **n** | **AR2 (%)** |
| Belo Horizonte | 387 | 380 | 98.2 | 370 | 95.6 |
| Boa Vista | 469 | 457 | 97.4 | 437 | 93.2 |
| Campo Grande | 362 | 355 | 98.1 | 340 | 93.9 |
| Cuiabá | 415 | 408 | 98.3 | 402 | 96.9 |
| Curitiba | 361 | 341 | 94.5 | 322 | 89.2 |
| Florianópolis | 370 | 364 | 98.4 | 356 | 96.2 |
| Goiânia | 356 | 329 | 92.4 | 317 | 89.0 |
| Macapá | 589 | 581 | 98.6 | 475 | 80.6 |
| Palmas | 474 | 469 | 98.9 | 444 | 93.7 |
| Salvador | 390 | 383 | 98.2 | 374 | 95.9 |
| São Luís | 521 | 513 | 98.5 | 506 | 97.1 |
| São Paulo | 452 | 440 | 97.3 | 426 | 94.2 |
| Teresina | 517 | 488 | 94.4 | 472 | 91.3 |
| Vitória | 423 | 414 | 97.9 | 394 | 93.1 |
| Total | 6,088 | 5,922 | 97.3 | 5,735 | 88.3 |

AR1=acceptance rate or the percentage of drivers who accepted to participate in the interview;

AR2= acceptance rate or the percentage of participants who agreed to take the breathalyzer test.
