# Supplementary material for: Estimate of the magnitude of risky and protective behaviors associated with road traffic injuries in capitals participating in the Life in Traffic Project of Brazil
Source: PLoS One. 2022 Oct 19;17(10):e0275537. doi: 10.1371/journal.pone.0275537 (PMC9581410; doi:10.1371/journal.pone.0275537)
Supplement: S1 Appendix — (DOCX) [file pone.0275537.s002.docx]

**Appendix 1.** Questionnaire

**ID participant: ___________.**

**Q1. Capital name**

( ) 1. Belo Horizonte.

( ) 2. Boa Vista.

( ) 3. Campo Grande.

( ) 4. Cuiabá.

( ) 5. Curitiba.

( ) 6. Florianópolis.

( ) 7. Goiânia.

( ) 8. Macapá

( ) 9. Palmas.

( ) 10. Salvador.

( ) 11. São Luís.

( ) 12. São Paulo.

( ) 13. Teresina.

( ) 14. Vitória.

Go to Q2.

**Q2. Interviewer: Record the type of vehicle.**

( ) 0. Cars/pickup truck/sport utility vehicle.

( ) 1. Motorcyclist

Go to Q3.

**Q3. Interviewer: Record the sex of the driver.**

( ) 0. Female.

( ) 1. Male.

Go to Q4.

**Q4. Interviewer: Record the date of birth (DD/MM/YYYY).**

|___|___|/|___|___|/|___|___|___|___|

Go to Q5.

**Q5. What was the last grade you studied?**

( ) 0. Never studied.

( ) 1. Elementary school incomplete.

( ) 2. Complete elementary school.

( ) 3. Incomplete high school.

( ) 4. Complete high school.

( ) 6. Complete higher education.

( ) 77. Doesn't know.

( ) 88. I didn't want to answer.

Go to Q6.

**Q6. What is your color or race?**

( ) 0. White.

( ) 1. Black.

( ) 2. Asian.

( ) 3. Mixed race.

( ) 4. Indigenuous.

( ) 88. I didn't want to answer.

Go to Q7.

**Q7. In the last 30 days, have you had at least one dose of alcoholic beverage?**

( ) 0. No.

( ) 1. Yes.

( ) 77. Doesn't know.

( ) 88. I didn't want to answer.

If 0, 77 or 88, skip to Q9. If 1, go to Q8.

**Q8. On any of these days when you consumed alcohol, did you drive right after drinking?**

( ) 0. No.

( ) 1. Yes.

( ) 77. Doesn't know.

( ) 88. I didn't want to answer.

Go to Q9.

**Q9. How often do you use your cell phone to make calls while driving (not including handsfree/bluetooth)?**

( ) 1. Always.

( ) 2. Almost always.

( ) 3. Sometimes.

( ) 4. Rarely.

( ) 5. Never.

( ) 77. Doesn't know.

( ) 88. I didn't want to answer.

Go to Q10.

**Q10. How often do you use your cell phone to send or read messages/whatsapp while driving?**

( ) 1. Always.

( ) 2. Almost always.

( ) 3. Sometimes.

( ) 4. Rarely.

( ) 5. Never.

( ) 77. Doesn't know.

( ) 88. I didn't want to answer.

If Q2=0, skip to Q11. If Q2=1, go to Q12.

**Q11. How often do you wear a seatbelt when driving?**

( ) 1. Always.

( ) 2. Almost always.

( ) 3. Sometimes.

( ) 4. Rarely.

( ) 5. Never.

( ) 77. Doesn't know.

( ) 88. I didn't want to answer.

Go to Q13.

**Q12. How often do you wear a helmet when riding a motorcycle?**

( ) 1. Always.

( ) 2. Almost always.

( ) 3. Sometimes.

( ) 4. Rarely.

( ) 5. Never.

( ) 77. Doesn't know.

( ) 88. I didn't want to answer.

Go to Q13.

**Q13. How often do you drive above the maximum speed on a road with a maximum speed of 50 km/h?**

( ) 1. Always.

( ) 2. Almost always.

( ) 3. Sometimes.

( ) 4. Rarely.

( ) 5. Never.

( ) 77. Doesn't know.

( ) 88. I didn't want to answer.

Go to Q14.

**Q14. How often do you usually pass the headlight/traffic/signal when it's red (not counting the dawn)?**

( ) 1. Always.

( ) 2. Almost always.

( ) 3. Sometimes.

( ) 4. Rarely.

( ) 5. Never.

( ) 77. Doesn't know.

( ) 88. I didn't want to answer.

Go to Q15.

To assess the consumption of alcohol in drivers, it is necessary to carry out the breathalyzer test. The test result will be confidential, and your test result will only be used for survey data. It will never be disclosed under any circumstances and will not be shared with any transit authority.

**Q15. Do you agree to take the breathalyzer test?**

( ) 0. No

( ) 1. Yes.

If 0, end survey; If 1, Go to Q16.

**Q16. Interviewer: record the identification number of the breathalyzer (located behind the device)**

|___|

Go to Q17.

**Q17. Interviewer: Record the test number that appears on the breathalyzer display.**

|___||___||___||___|

Go to Q18.

**Q18. Interviewer, record the concentration shown on the breathalyzer test.**

|___|___|___|

End survey.
